# MI-GWAS: a SAS platform for the analysis of inherited and maternal genetic effects in genome-wide association studies using log-linear models

**DOI:** 10.1186/1471-2105-12-117

**Published:** 2011-04-22

**Authors:** AJ Agopian, Laura E Mitchell

**Affiliations:** 1 Human Genetics Center, Division of Epidemiology, Human Genetics and Environmental Sciences, The University of Texas School of Public Health, 1200 Herman Pressler Dr., Houston, TX 77030, USA

## Abstract

**Background:**

Several platforms for the analysis of genome-wide association data are available. However, these platforms focus on the evaluation of the genotype inherited by affected (i.e. case) individuals, whereas for some conditions (e.g. birth defects) the genotype of the mothers of affected individuals may also contribute to risk. For such conditions, it is critical to evaluate associations with both the maternal and the inherited (i.e. case) genotype. When genotype data are available for case-parent triads, a likelihood-based approach using log-linear modeling can be used to assess both the maternal and inherited genotypes. However, available software packages for log-linear analyses are not well suited to the analysis of typical genome-wide association data (e.g. including missing data).

**Results:**

An integrated platform, Maternal and Inherited Analyses for Genome-wide Association Studies **(**MI-GWAS) for log-linear analyses of maternal and inherited genetic effects in large, genome-wide datasets, is described. MI-GWAS uses SAS and LEM software in combination to appropriately format data, perform the log-linear analyses and summarize the results. This platform was evaluated using existing genome-wide data and was shown to perform accurately and relatively efficiently.

**Conclusions:**

The MI-GWAS platform provides a valuable tool for the analysis of association of a phenotype or condition with maternal and inherited genotypes using genome-wide data from case-parent triads. The source code for this platform is freely available at http://www.sph.uth.tmc.edu/sbrr/mi-gwas.htm.

## Background

Candidate gene, and more recently, genome-wide association (GWA) studies have been used to identify associations between several complex diseases and the genotype of affected individuals (i.e. cases) [1-6]. However, for some phenotypes (e.g. birth defects, perinatal outcomes, pediatric cancers), the maternal genotype may also directly contribute to risk, via an effect on the *in utero *environment [7]. However, despite increasing recognition of the importance of maternal genetic effects in genetic epidemiology studies [8-17], nearly all GWA studies to date have ignored maternal genetic effects [18], which could partially explain why GWA studies have not identified the majority of the genetic contribution to common diseases. Failure to account for maternal genetic effects in previous studies may be due both to lack of appropriate data (i.e. maternal DNA is not collected in traditional case-control studies) and lack of a readily available platform for performing analyses that account for maternal genetic effects using typical GWA data.

The most common study designs used in GWA studies include the case-control and case-parent triad designs. Though the case-control approach has been used more frequently, distinguishing between maternal and inherited genetic effects using this study design requires the addition of samples from mothers of both cases and controls, resulting in increased genotyping costs [7]. Moreover, maternal DNA has not been collected for most existing case-control studies. By contrast, maternal genetic effects can be directly assessed in existing data from case-parent triad GWA studies. However, in the majority of these studies, data have been analyzed using the transmission disequilibrium test (TDT) [19,20], which does not assess maternal genetic effects.

The most commonly used method for assessing maternal and inherited genetic effects using case-parent triad data is a log-linear, likelihood-based approach [21,22]. In addition to evaluating maternal genetic effects, this approach has the advantage (over the TDT) of providing estimates of effect size and, similar to the TDT, does not require the assumption of Hardy-Weinberg equilibrium [21-24]. Further, using this approach, data from incomplete triads can be included in the analysis by use of the expectation-maximization (EM) algorithm [23]. These log-linear analyses can be conducted using standard software (e.g. SAS^®^, SAS Institute Inc.), but, when data from incomplete triads are included, require programming of the EM, which is cumbersome. The specialized program, LEM [25,26], can also be used to conduct these analyses, and has the advantage over other programs (e.g. SAS) in that it does not require programming of the EM and can be easily programmed to explore a variety of additional types of effects (e.g., gene-gene interaction, gene-environment interaction). However, because LEM requires individual data and program files for each SNP, it is not feasible to conduct a GWA study analysis using LEM as a stand-alone program.

To address the need for an efficient approach for analyzing maternal and inherited genetic effects using GWA data from case-parent triad studies, a novel computational platform was created. This platform uses SAS to prepare the data in an LEM-compatible format, calls LEM to evaluate each SNP, and extracts and summarizes relevant data from the LEM output files. The performance of this platform was evaluated using existing, case-parent triad GWA data.

## Implementation

### Log-linear Modeling

The theoretical distribution (assuming Mendelian inheritance and mating symmetry) of case-parent triad genotype data (defined by the combination of father, mother, and child genotypes) can be fitted to the observed triad counts by maximum likelihood using the following log-linear model:

The values of E(*n*_F,M,C_) correspond to the expected count of each genotype combination (i.e., father, mother, and child genotypes). The γ_j _term stratifies on parental mating type (i.e., each combination of possible parental genotypes), and I is an indicator variable that equals 0, 1, or 2, corresponding to the number of high-risk alleles present in the child's or mother's genotype (C and M respectively). The offset, *w*_F,M,C_, accounts for the heterozygous genotype being twice as likely as either homozygous genotype in offspring of double heterozygous matings (assuming Mendelian transmission). Under this model, the risk corresponding to a genotype with one copy of the "high-risk" allele compared to no copies can be estimated by exp(β_1_) for the inherited genotype and exp(α_1_) for the maternal genotype, and the risk corresponding to a genotype with two copies compared to no copies can be estimated by exp(β_2_) for the inherited genotype and exp(α_2_) for the maternal genotype. The significance of the inherited genetic effect can be evaluated using a two degree of freedom likelihood ratio test (LRT) to compare the log-likelihood of the full model (i.e. Model 1) to that of a reduced model in which β_1 _= β_2 _= 0. The null hypothesis for this test is that conditional on parental mating type, the distribution of affected offspring agrees with Mendelian expectation. Similar to the TDT, this LRT provides a test of linkage in the presence of linkage disequilibrium that is not vulnerable to confounding due to population stratification [21,27]. Likewise, the significance of the maternal genetic effect can be evaluated by using the LRT to compare the log-likelihood of the full model to that of a reduced model in which α_1 _= α_2 _= 0. The null hypothesis for this test is that reciprocal parental mating types (e.g. Aa × AA and AA × Aa) occur at the same frequency among parents of affected individuals [21-23]. This LRT is vulnerable to a specific form of population stratification that violates the underlying assumption of mating symmetry. Mating asymmetry occurs when reciprocal mating types for a given allele do not occur with equal frequency in the population (e.g. Aa × AA > AA × Aa). The potential for this type of bias can be limited by restricting analyses to matings from the same race and ethnicity [7]. Data from incomplete triads can also be included in these analyses by use of the EM algorithm.

Model 1 is a general (i.e. unrestricted) model, in which no constraints are placed on the relationships between alleles (i.e. no constraints on β_1 _and β_2 _or α_1 _and α_2_). However, in some circumstances power can be increased by imposing constraints on the general model. For example, a linear constraint can be imposed upon the full model for the genetic effect being tested, leaving the terms for the other genetic effect unconstrained. This allows for a one degree of freedom LRT. The use of a linear constraint has been shown to perform well under a variety of circumstances [7,21-23,28].

### LEM Software

The log-linear modeling approach with EM can be implemented using the LEM program, which can be downloaded at: http://spitswww.uvt.nl/~vermunt/#Software. LEM requires individual data and program files for each association test. Thus, for each SNP, it requires one data file and four program files: one for the full model with a linear constraint imposed for inherited effects, one for the full model with a linear constraint imposed for maternal effects, one for the reduced model to test for inherited effects, and one for the reduced model to test for maternal effects. Further, LEM data files must be formatted so that each row contains genotypes for the three members of the triad, whereas it is common for genome-wide genotype data to be outputted as a single file with one individual per row and one allele per column (e.g. PLINK [29] format) (Table 1).

**Table 1:** Comparison of PLINK (A) and LEM (B) data format and example data for three hypothetical case-parent triads

(A)															
**PLINK Genotype Data for 5 SNPs**															

**Family Number**	**Individual ID**	**Paternal ID**	**Maternal ID**	**Sex**^**a**^	**Phenotype**^**b**^	**SNP 1**^**c**^		**SNP 2**^**c**^		**SNP 3**^**c**^		**SNP 4**^**c**^		**SNP 5**^**c**^	

1001	1001-A	1001-C	1001-B	1	1	1	1	0	0	1	1	1	1	1	2
1001	1001-B	0	0	2	0	1	1	1	1	1	2	1	1	1	1
1001	1001-C	0	0	1	0	1	1	1	2	1	1	1	1	0	0
1002	1002-A	1002-C	1002-B	2	1	0	0	2	2	1	2	1	1	1	2
1002	1002-B	0	0	2	0	2	2	1	2	0	0	1	1	1	1
1002	1002-C	0	0	1	0	1	1	2	2	1	1	1	2	2	2
1003	1003-A	0	1003-B	2	1	1	2	1	2	1	1	1	1	1	1
1003	1003-B	0	0	2	0	1	1	1	2	1	1	0	0	1	1

															
**(B)**															

**LEM Data File for SNP 1**															

**Mother's Genotype**^**d**^			**Father's Genotype**^**d**^			**Case's Genotype**^**d**^									

1			1			1									
3			1			0									
1			0			2									

^a ^1 = male, 2 = female

^b ^1 = unaffected, 2 = affected

^c ^0 = allele missing, 1 = allele 1, 2 = allele 2

^d ^0 = genotype missing, 1 = no high-risk alleles present, 2 = one high-risk alleles present, 3 = two high-risk alleles present

### Maternal and Inherited Analyses for Genome-wide Association Studies (MI-GWAS)

SAS version 9.2 was used to develop a platform for the efficient analysis of maternal and inherited genetic effects in GWA data, using LEM to apply the log-linear modeling likelihood-based approach. This platform is called Maternal and Inherited Analyses for Genome-wide Association Studies (MI-GWAS) and is freely available at http://www.sph.uth.tmc.edu/sbrr/mi-gwas.htm.

Briefly, MI-GWAS:

1. Reads data from a single PLINK ped file that contains a separate row of data for each triad member. Each row contains all genotype data for a single individual, and each genotype is coded as two alleles (i.e. two columns of data per genotype). By default, MI-GWAS treats the allele coded as "2" under the Illumina 1/2 allele coding system as the high-risk allele.

2. Converts the PLINK data into one LEM data file per SNP, with a single row of genotype data for each triad, with genotypes in the order of mother, father, and child. These genotypes are coded as 0, 1, or 2, for number of high-risk alleles present (i.e. one column of data per genotype).

3. Creates LEM program files for each SNP - one program file per model.

4. Calls LEM to run the relevant analyses (i.e. full and reduced models) for each SNP.

5. Extracts relevant output (i.e. log-likelihood values, relative risks and corresponding standard errors) from LEM.

6. Calculates relevant LRTs and associated p-values.

7. Computes 95% confidence intervals (CIs) for all relative risks.

8. Merges results with the PLINK marker map file, which includes chromosome number and rs number for each SNP.

9. Sorts results by the LRT p-value, separately for the inherited and maternal genotype, and outputs two corresponding data files.

10. Sorts results by relative risk estimates, separately for the inherited and maternal genotype, and outputs two corresponding data files.

**Figure 1:**
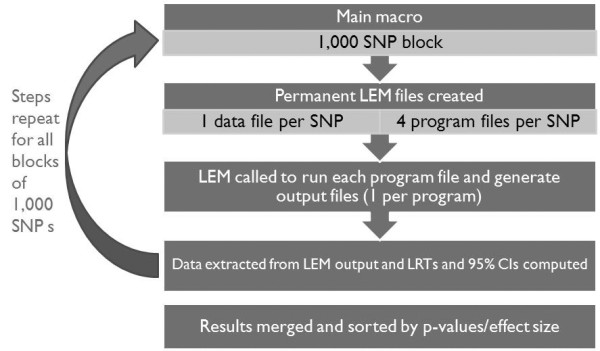
**Summary of MI-GWAS platform structure, displaying steps preformed on subsets of 1,000 SNPs at a time**.

To increase computation efficiency, repeating sections of code (i.e. macros) are used to split these steps into consecutive blocks that process subsets of SNPs (by default 1,000). Each consecutive subset of SNPs is processed separately, and after processing of the last subset is complete, all results are merged. A visual overview of the structure of these steps of the platform is provided in Figure 1.

The use of SAS, a popular statistical software package, allows for flexibility in the MI-GWAS platform with simple programming changes. For example, the user can specify a range of successive SNPs to analyze rather than running all SNPs at once. This allows the user to split the analysis between multiple runs or between multiple computers. The program also includes code that can be modified to allow the user to specify specific individuals, triads, or SNPs to be excluded from the analysis. Further, the log-linear models can be modified to accommodate other family-based association designs (e.g. study designs that incorporate grandparents' genotypes [7]), other relationships between alleles (e.g. dominant inheritance), and to include other effects such as gene-gene and gene-environment interactions and parent of origin effects. By default, a linear constraint is imposed for the effect that is being tested (e.g. inherited genotype) and no constraint is imposed for the other effect (e.g. maternal genotype). The platform can also process imputed data that has been converted to PLINK format. Though by default MI-GWAS processes PLINK-formatted genotype data with Illumina 1/2-based allele coding, the SAS code can be modified to process genotype data that are in other formats.

### Evaluation

To validate the MI-GWAS platform and evaluate its performance, it was used to analyze a large, unpublished GWA study dataset derived from case-parent triads ascertained through cases with a conotruncal heart defect. The subject recruitment methods for this study have been previously described [17]. Briefly, case-parent triads were recruited through the Cardiac Center at the Children's Hospital of Philadelphia (CHOP) from 1997-2007 and all participants provided informed consent under a protocol approved by the Institutional Review Boards for the Protection of Human Subjects. Cases had a nonsyndromic, classic conotruncal defect (i.e. tetalogy of Fallot, D-transposition of the great arteries, double outlet right ventricle, truncus arteriosus or interrupted aortic arch) or a related malformation (i.e. perimembranous or posterior malalignment type ventricular septal defect or an isolated aortic arch anomaly). The genotype data were generated from blood or saliva samples using the Illumina HumanHap550 or Human610-Quad BeadChip Platforms at the Center for Applied Genomics at CHOP. Only SNPs present on both platforms were analyzed.

To verify MI-GWAS results for inherited genetic effects using the CHOP dataset, selected SNPs were evaluated for complete triads using both PLINK and MI-GWAS and the chi-square values for the TDT and LRT, respectively, were compared. (The TDT approach under PLINK cannot assess maternal genetic effects and does not incorporate data from incomplete triads into evaluations of inherited genetic effects.) To verify MI-GWAS results for maternal genetic effects using this dataset, selected SNPs were evaluated using both MI-GWAS and LEM run outside of MI-GWAS, and the resulting LRT p-values were compared. In addition, MI-GWAS was used to replicate the findings of a candidate gene study conducted in this study population, using LEM run outside of MI-GWAS [17]. To be consistent with the previous analyses, MI-GWAS was modified to use an unrestricted model of inheritance for both genotypes (i.e. a two degree of freedom LRT), for the latter analysis.

## Results

The evaluation dataset included data on 530,350 SNPs from 837 case-parent triads (497 complete triads and 340 triads with one or two members missing). To confirm MI-GWAS results for the test of inherited genetic effect, MI-GWAS was used to analyze all SNPs in the subset of complete triads, and chi-square values for the LRT were compared to TDT chi-square values obtained using PLINK to analyze the same data. The chi-square values for the LRTs of inherited genetic effects generated by MI-GWAS were quite similar to the TDT chi-square values generated using PLINK (Table 2). There were subtle differences in the chi-square values for some of the most significant SNPs, likely due to differences between the two platforms in rounding and/or how numbers are stored (e.g., floating point representation). In addition, identical results were obtained when program and data files generated by MI-GWAS were analyzed using the MI-GWAS platform and LEM run outside of MI-GWAS (data not shown). Finally, the results for maternal and inherited genetic effects from a previously published candidate gene study from the same study population [17] were replicated using MI-GWAS (data not shown).

**Table 2:** Comparison of chi-square values from the PLINK TDT and MI-GWAS LRT for inherited genetic effects for a randomly selected set of SNPs on chromosome one and most significant autosomal SNPs.

SNP	PLINK TDT chi-square value	MI-GWAS log-linear modeling chi-square value	**Percent Difference**^**a**^
Chromosome 1 SNPs			
SNP 1	0.02	0.02	0.00
SNP 2	2.22	2.22	0.00
SNP 3	0.91	0.91	0.00
SNP 4	0.09	0.09	0.00
SNP 5	0.24	0.24	0.00
SNP 6	1.58	1.58	0.00
SNP 7	2.97	2.98	0.00
SNP 8	1.22	1.22	0.00
Most significant autosomal SNPs			
SNP 9	22.34	24.06	0.08
SNP 10	22.29	22.57	0.01
SNP 11	21.48	21.64	0.01
SNP 12	20.78	21.02	0.01
SNP 13	19.56	19.77	0.01

^a ^Absolute difference between PLINK chi-square value and MI-GWAS chi-square value divided by PLINK chi-square value

As expected, MI-GWAS running times were somewhat faster on computers with better specifications. For the same 60,000 SNPs, running times ranged from 11 hours 35 minutes to 22 hours 48 minutes for four computers with differing specifications (Table 3).

**Table 3:** Running times for the analysis of the same 60,000 SNPs using MI-GWAS on four computers with differing specifications

Machine specifications	Running Time
Intel Core 2 Quad CPU Q9550, 2.83 GHz, 3.21 Gb of RAM	11 hours 35 minutes
Pentium 4 CPU, 3.00 GHz, 2.00 Gb of RAM	20 hours 52 minutes
Pentium D CPU, 3.20 GHz, 1.99 Gb of RAM	21 hours 24 minutes
Intel Xeon CPU E5540, 2.53 GHz, 6.00 Gb of RAM	22 hours 48 minutes

## Discussion

Computational platforms that can evaluate maternal as well as inherited genetic effects in genome-wide data from case-parent triads have not been previously described. By automating the implementation of such analyses, MI-GWAS provides such a platform. Comparison of results from MI-GWAS and LEM run outside of MI-GWAS, as well as results from MI-GWAS and PLINK, indicate that this platform performs as intended. Further, MI-GWAS performs relatively efficiently. From the observed running times (Table 3), it can be inferred that on a single average modern consumer computer, it may take approximately one week to run a GWA study analysis of ~500,000 SNPs using MI-GWAS. Such an analysis may run overnight if split between around eight average consumer computers.

The MI-GWAS platform has the advantage of using readily available software (i.e. SAS and LEM) and reading a common GWA data input format (i.e. PLINK format). Further, unlike the TDT approach under PLINK, analysis under MI-GWAS uses a log-linear approach that provides estimates of effect size, allows use of data from incomplete triads [21-23], and, most importantly, allows estimation of the significance of maternal effects in addition to inherited effects.

## Conclusions

For some conditions, maternal genetic effects may influence the risk of disease in offspring via an effect on the in utero environment. However, maternal genetic effects have not been widely evaluated in GWA studies, at least partially due to lack of a platform designed to analyze maternal genetic effects using GWA data from case-parent triads. The application of the MI-GWAS platform to GWA analyses expands the types of genetic effects that can be evaluated with triad GWA data, which may lead to new insights regarding the etiology of common diseases. Future developments of the MI-GWAS platform will involve improving the efficiency of the platform, and incorporating analyses of additional types of effects (e.g. parental imprinting, interactions).

## Availability and Requirements

Project name: MI-GWAS (Maternal and Inherited Analyses for Genome-wide Association Studies)

Project home page: http://www.sph.uth.tmc.edu/sbrr/mi-gwas.htm

Operating system(s): Platform independent

Programming language: SAS^®^

Other requirements: SAS^® ^release 9.2, LEM release 1.0 (freely available at http://spitswww.uvt.nl/~vermunt/#Software)

Licence: None for MI-GWAS

Any restrictions to use by non-academics: None for MI-GWAS

## Authors' contributions

AJA developed and tested the MI-GWAS platform, and wrote the manuscript. LEM conceived the study, provided input on the design and testing of the MI-GWAS platform and helped to draft and edit the manuscript. Both authors read and approved the final manuscript.

## References

[B1] Genome-wide association study of 14,000 cases of seven common diseases and 3,000 shared controls Nature 2007 447 661 678 10.1038/nature05911 17554300 PMC2719288

[B2] Rioux JD Xavier RJ Taylor KD Silverberg MS Goyette P Huett A Green T Kuballa P Barmada MM Datta LW Genome-wide association study identifies new susceptibility loci for Crohn disease and implicates autophagy in disease pathogenesis Nat Genet 2007 39 596 604 10.1038/ng2032 17435756 PMC2757939

[B3] Gudmundsson J Sulem P Manolescu A Amundadottir LT Gudbjartsson D Helgason A Rafnar T Bergthorsson JT Agnarsson BA Baker A Genome-wide association study identifies a second prostate cancer susceptibility variant at 8q24 Nat Genet 2007 39 631 637 10.1038/ng1999 17401366

[B4] Grant SF Qu HQ Bradfield JP Marchand L Kim CE Glessner JT Grabs R Taback SP Frackelton EC Eckert AW Follow-up analysis of genome-wide association data identifies novel loci for type 1 diabetes Diabetes 2009 58 290 295 10.2337/db08-1022 18840781 PMC2606889

[B5] Hakonarson H Qu HQ Bradfield JP Marchand L Kim CE Glessner JT Grabs R Casalunovo T Taback SP Frackelton EC A novel susceptibility locus for type 1 diabetes on Chr12q13 identified by a genome-wide association study Diabetes 2008 57 1143 1146 10.2337/db07-1305 18198356

[B6] Easton DF Pooley KA Dunning AM Pharoah PD Thompson D Ballinger DG Struewing JP Morrison J Field H Luben R Genome-wide association study identifies novel breast cancer susceptibility loci Nature 2007 447 1087 1093 10.1038/nature05887 17529967 PMC2714974

[B7] Mitchell LE Weinberg CR Evaluation of offspring and maternal genetic effects on disease risk using a family-based approach: the "pent" design Am J Epidemiol 2005 162 676 685 10.1093/aje/kwi249 16093287

[B8] Boyles AL Wilcox AJ Taylor JA Shi M Weinberg CR Meyer K Fredriksen A Ueland PM Johansen AM Drevon CA Oral facial clefts and gene polymorphisms in metabolism of folate/one-carbon and vitamin A: a pathway-wide association study Genet Epidemiol 2009 33 247 255 10.1002/gepi.20376 19048631 PMC2677659

[B9] van der Put NM Steegers-Theunissen RP Frosst P Trijbels FJ Eskes TK van den Heuvel LP Mariman EC den Heyer M Rozen R Blom HJ Mutated methylenetetrahydrofolate reductase as a risk factor for spina bifida Lancet 1995 346 1070 1071 10.1016/S0140-6736(95)91743-8 7564788

[B10] Mitchell LE Long J Garbarini J Paluru P Goldmuntz E Variants of folate metabolism genes and risk of left-sided cardiac defects Birth Defects Res A Clin Mol Teratol 88 48 53 19777601 10.1002/bdra.20622 PMC2860744

[B11] Reutter H Birnbaum S Mende M Almeida de Assis N Hoffmann P Lacava AD Herms S Braumann B Scheer M Lauster C Transforming growth factor-beta receptor type 1 (TGFBR1) is not associated with non-syndromic cleft lip with or without cleft palate in patients of Central European descent Int J Pediatr Otorhinolaryngol 2009 73 10 1334 8 10.1016/j.ijporl.2009.06.004 19586667

[B12] Mills JL Molloy AM Parle-McDermott A Troendle JF Brody LC Conley MR Cox C Pangilinan F Orr DJ Earley M Folate-related gene polymorphisms as risk factors for cleft lip and cleft palate Birth Defects Res A Clin Mol Teratol 2008 82 636 643 10.1002/bdra.20491 18661527 PMC2670560

[B13] Little J Gilmour M Mossey PA Fitzpatrick D Cardy A Clayton-Smith J Hill A Duthie SJ Fryer AE Molloy AM Scott JM Folate and clefts of the lip and palate--a U.K.-based case-control study: Part II: Biochemical and genetic analysis Cleft Palate Craniofac J 2008 45 428 438 10.1597/06-151.1 18616362

[B14] Ehn NL Cooper ME Orr K Shi M Johnson MK Caprau D Dagle J Steffen K Johnson K Marazita ML Evaluation of fetal and maternal genetic variation in the progesterone receptor gene for contributions to preterm birth Pediatr Res 2007 62 630 635 10.1203/PDR.0b013e3181567bfc 17805208 PMC2734951

[B15] Jensen LE Hoess K Mitchell LE Whitehead AS Loss of function polymorphisms in NAT1 protect against spina bifida Hum Genet 2006 120 52 57 10.1007/s00439-006-0181-6 16680433

[B16] Infante-Rivard C Vermunt JK Weinberg CR Excess transmission of the NAD(P)H:quinone oxidoreductase 1 (NQO1) C609T polymorphism in families of children with acute lymphoblastic leukemia Am J Epidemiol 2007 165 1248 1254 10.1093/aje/kwm022 17332311 PMC2080583

[B17] Goldmuntz E Woyciechowski S Renstrom D Lupo PJ Mitchell LE Variants of folate metabolism genes and the risk of conotruncal cardiac defects Circ Cardiovasc Genet 2008 1 126 132 10.1161/CIRCGENETICS.108.796342 20031554 PMC3035562

[B18] Hindorff LA Junkins HA Mehta JP Manolio TA A Catalog of Published Genome-Wide Association Studies 2010

[B19] Spielman RS Ewens WJ The TDT and other family-based tests for linkage disequilibrium and association American Journal of Human Genetics 1996 59 983 989 8900224 PMC1914831

[B20] Benyamin B Visscher PM McRae AF Family-based genome-wide association studies Pharmacogenomics 2009 10 181 190 10.2217/14622416.10.2.181 19207019

[B21] Weinberg CR Wilcox AJ Lie RT A log-linear approach to case-parent-triad data: assessing effects of disease genes that act either directly or through maternal effects and that may be subject to parental imprinting Am J Hum Genet 1998 62 969 978 10.1086/301802 9529360 PMC1377041

[B22] Wilcox AJ Weinberg CR Lie RT Distinguishing the effects of maternal and offspring genes through studies of "case-parent triads" Am J Epidemiol 1998 148 893 901 9801020 10.1093/oxfordjournals.aje.a009715

[B23] Weinberg CR Allowing for missing parents in genetic studies of case-parent triads Am J Hum Genet 1999 64 1186 1193 10.1086/302337 10090904 PMC1377843

[B24] Starr JR Hsu L Schwartz SM Assessing maternal genetic associations: a comparison of the log-linear approach to case-parent triad data and a case-control approach Epidemiology 2005 16 294 303 10.1097/01.ede.0000158223.98649.eb 15824543

[B25] Vermunt JK LEM: a general program for the analysis of categorical data 1997 Tilberg University

[B26] van Den Oord EJ Vermunt JK Testing for linkage disequilibrium, maternal effects, and imprinting with (In)complete case-parent triads, by use of the computer program LEM Am J Hum Genet 2000 66 335 338 10.1086/302708 10631165 PMC1288341

[B27] Spielman RS McGinnis RE Ewens WJ Transmission test for linkage disequilibrium: the insulin gene region and insulin-dependent diabetes mellitus (IDDM) Am J Hum Genet 1993 52 506 516 8447318 PMC1682161

[B28] Starr JR Hsu L Schwartz SM Performance of the log-linear approach to case-parent triad data for assessing maternal genetic associations with offspring disease: type I error, power, and bias American Journal of Epidemiology 2005 161 196 204 10.1093/aje/kwi021 15632270

[B29] Purcell S Neale B Todd-Brown K Thomas L Ferreira MA Bender D Maller J Sklar P de Bakker PI Daly MJ Sham PC PLINK: a tool set for whole-genome association and population-based linkage analyses Am J Hum Genet 2007 81 559 575 10.1086/519795 17701901 PMC1950838

